# Case of Extensive Fracture of the Skull, and Perforation of the Longitudinal Sinus—Operation and Recovery

**Published:** 1849-10

**Authors:** W. C. Cole

**Affiliations:** Detroit, Michigan


Art. II.—*Case of Extensive Fracture of the Skull, and Perforation of the Longitudinal Sinus—Operation and Recovery.* By W. C. Cole, M. D., of Detroit, Michigan.

Arad F., æt. 19, resident of Seneca county, Ohio, was struck down early in the morning of the 4th day of February, 1848, by a red-oak limb, four and a half inches in diameter, and twenty feet long, falling horizontally, from a height of fifty feet, hitting him upon the top and back part of the head, transversely, as he was standing erect, having no warning of the coming blow, and breaking from the centre of the limb, a piece six feet long, and extensively fracturing the superior and posterior portions, of the parietal, and the superior portion of the occipital bones.

Drs. Pierce and Sparks were immediately called to the case. They requesting my presence, I was accordingly sent for. On my arrival, towards evening, I found the patient in the following condition : Laying upon his back, and at times somewhat restless ; respiration laborious and slow; pulse fifty per minute and heavy ; extremities cold ; face expressive of pain and anxiety ; eyes injected, and pupils largely dilated, yet sluggishly sensible to light; tongue white and blanched ; incoherent muttering ; could be partially roused, yet could give no answers when questioned ; when head was handled, moaned and was restless; would also throw his hands over his head; great tumefaction of the scalp covering the upper and back part of the head.

He was immediately bled to the extent of twenty ounces, without materially effecting the pulse or respiration. Hot applications were made to the feet, the head ed, and cold applied to it.

I now recommended the operation, as there was, beyond a doubt, extensive depression of the bones of the cranium.

Here a truce was sounded, for family consultation, which became somewhat protracted, in consequence of a discrepancy of opinion between the attending physicians, previous to my arrival. The one, an old practitioner, gave it as his opinion, that there was no fracture or depression of the skull. The other, a young physician, and former pupil of mine, had expressed a contrary opinion. Our dissenting friend, however, soon acquiesced, and the friends of the patient gave their consent, and desired the operation, as the last chance left, to save the life of their brother, (his parents being absent,) and that chance a very doubtful one, from the locality and extent of the injury sustained.

Accordingly, after shaving the hair from all that portion of the scalp, occupied by the tumefaction, the operation was commenced, about sunset, by making a crucial incision of five inches in extent, across the tumor, and raising the flaps, the pericranium was found largely detached from the superior posterior angle of the right parietal bone. The borders of both parietals, forming the posterior third of the sagittal suture fractured and depressed—the right more than the left. The occipital bone was also fractured and depressed along its lambdoidal edge, for three inches, and extending an inch and a half into its substance. The Trephine was first set, with its centre pin just upon the sound edge of the occipital bone, and a perforation made. It was next applied to the left parietal, which was also perforated. Now connecting the two perforations, with a "Hay's saw," crossing the lambdoidal suture, a large portion of depressed bone was removed, from the line made with the "Hay's saw" to the sagittal suture. This being accomplished, the remaining fractured portion of the occipital was readily removed by the aid of the bone-nippers and forceps.

There was now only left to be elevated, and some pieces removed, of an ugly fracture and depression of the right, together with a small portion of the opposing edge of the left parietal, extending over the longitudinal sinus. To effect this, the trephine was placed on the sound border of the right parietal bone, (it being the part denuded of its pericranium,) but immediately on making pressure, to bring the saw in contact with the bone, a quantity of blood made its exit from the first perforations. When the pressure was removed, the blood ceased to flow, but as soon as reapplied, the flowing recommenced, and continued, so long as pressure was kept up.

The perforation, however, was soon accomplished so nearly, that the piece was broken out with the elevator. This being done, I proceeded to remove and elevate the remaining fractured and depressed portions of bone, composing the sagittal suture from both parietals, and on grasping with the forceps, a small spicula of bone, which was driven inward, a continuous jet of venous blood, proceeding from under the edge of the left parietal, followed its removal. This was imperfectly suppressed by the finger of an assistant, until the remaining loose pieces of bone could be removed and the firm depressions elevated, which being done rather hurriedly, as I confess I now considered the case hopeless, a compress of muslin was folded of sufficient thickness, and laid over the bleeding sinus, so that when the flaps were brought together, it effectually suppressed the hemorrhage which had been so profuse, during the few moments occupied in closing the operation, that the patient became almost pulseless. After closing the wound, by stitching the corners of the flaps together, and applying suitable bandages, I left the patient for the night, with Dr. Sparks, assuring the friends that he would die.

Dr. Pierce continued to attend the case, until, contrary to my most positive prognosis, he recovered, with every faculty which he had ever possessed; perfect in every respect, except the small handful of bone which I had relieved him of, and still have in my possession.

I regret that the daily minutes of this case were not kept. The wound was dressed in one week from the operation, and the compress removed. Slight spasms occurred occasionally, for some two weeks, until suppuration was fairly established, and as soon as the exfoliation of the external table of the right parietal, which was denuded of its pericranium was completed, healthy granulations sprang up, and the case went kindly on to a most favorable termination.

*Remarks.*—In reflecting upon this case, it is evident that the patient owed his recovery to the great quantity of blood which escaped from the wounded sinus, thereby holding the reacting powers of nature in check, until the low grade of inflammation which was set up, (as without inflammation no recuperative effect could result) sufficiently agglutinated together the edges of the wound in the sinus, to withstand the force of the circulation, when it became vehement, which was kept somewhat restrained, by cold applications.

